# Iatrogenic lumbosacral infiltration with petroleum (hydrodesulfurized heavy) with secondary intrathecal distribution—a case report

**DOI:** 10.1007/s00701-023-05505-w

**Published:** 2023-02-03

**Authors:** Fernando Muruato-Araiza, Simon Oekenpöhler, Nana-Maria Wagner, Petra Förster, Nils Warneke, Markus Holling, Manoj Mannil, Oliver Martin Grauer, Walter Stummer, Benjamin Brokinkel

**Affiliations:** 1 Department of Neurosurgery, University Hospital Münster, North Rhine-Westphalia, Albert-Schweitzer-Campus 1, Gebäude A1, 48149 Münster, Germany; 2 Department of Trauma-, Hand- and Reconstructive Surgery, University Hospital Münster, North Rhine-Westphalia, Münster, Germany; 3 Department of Anaesthesiology, Intensive Care and Pain Medicine, University Hospital Münster, North Rhine-Westphalia, Münster, Germany; 4 Poison Center Bonn, Children’s University Hospital, North Rhine-Westphalia, Bonn, Germany; 5 Institute for Clinical Radiology, University Hospital Münster, North Rhine-Westphalia, Münster, Germany; 6 Department of Neurology, University of Münster, North Rhine-Westphalia, Münster, Germany

**Keywords:** Intoxication, Lavage, Myeloencephalopathy, Petroleum, Ventricular drain

## Abstract

Petroleum is commonly used as a solvent, and primary intrathecal administration or secondary diffusion and subsequent clinical management has not been reported. We report the case of a male patient with intrathecal petroleum diffusion following accidental lumbar infiltration. After the onset of secondary myeloencephalopathy with coma and tetraparesis, continuous cranio-lumbar irrigation using an external ventricular and a lumbar drain was established. Cranial imaging revealed distinct supra- and infratentorial alterations. The patient improved slowly and was referred to rehabilitation. Intrathecal petroleum leads to myeloencephalopathy and continuous cranio-lumbar irrigation might be a safe treatment option.

## Introduction

Petroleum (hydrodesulfurized heavy) predominantly consists of fluid hydrocarbon derived from mineral oil is extremely lipophile and therefore commonly used for dissolving or industrial cleaning. Parenteral intake, e.g., in suicide attempts, has been described in few reports, and usually induces extensive necrosis with a partially fatal outcome [2, 5, 7, 10]. However, the effects after intrathecal administration of petroleum have not been published yet. As the first report so far, we here describe a case of accidental lumbosacral petroleum infiltration with subsequent intrathecal distribution, the clinical course as well as the management and outcome of the patient.

## Methods and materials

We here describe the clinical course and management of a patient after accidental infiltration of petroleum.

## Results

**Fig. 1:**
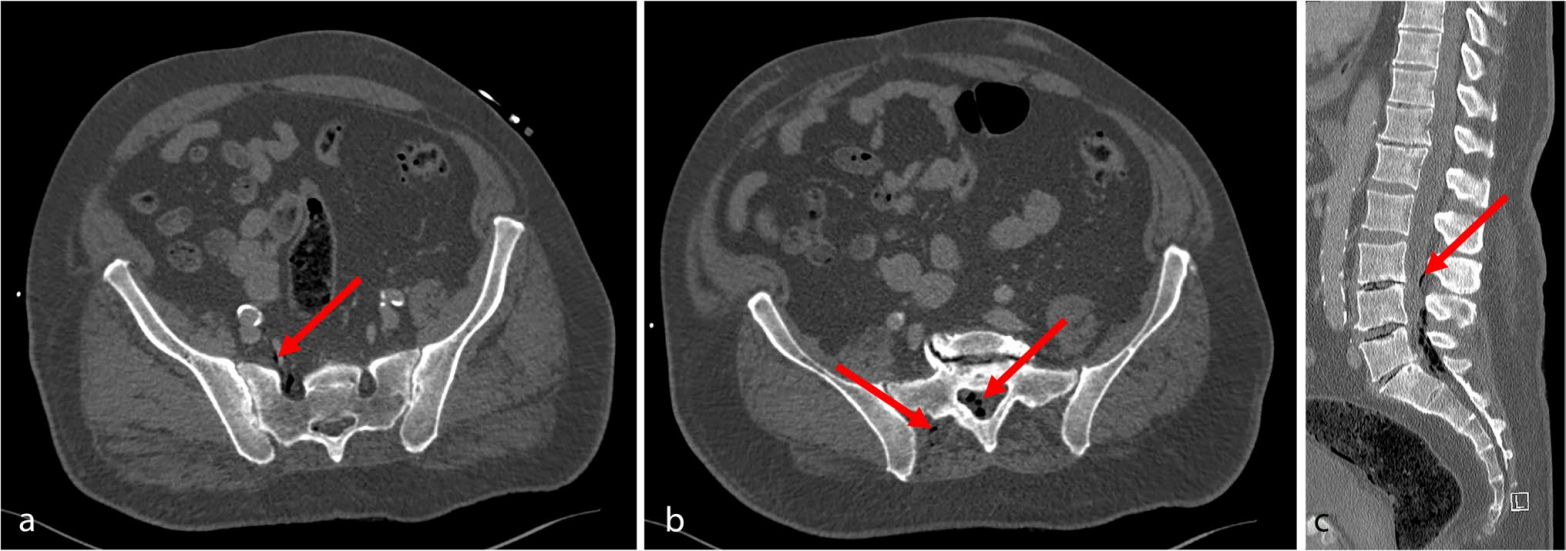
Computed tomography (CT) imaging 2 h after petroleum exposition. Axial (**a** and **b**) and sagittal (**c**) imaging reveal intramuscular gas distribution (red arrows), along the nerve roots (**a**) and, likely, between the cauda equina fibers (**b**) and ascendence up to the level of L3 (**c**), suggestive for intrathecal diffusion. Due to obesity of the patient, primary intrathecal injection was considered unlikely

**Fig. 2:**
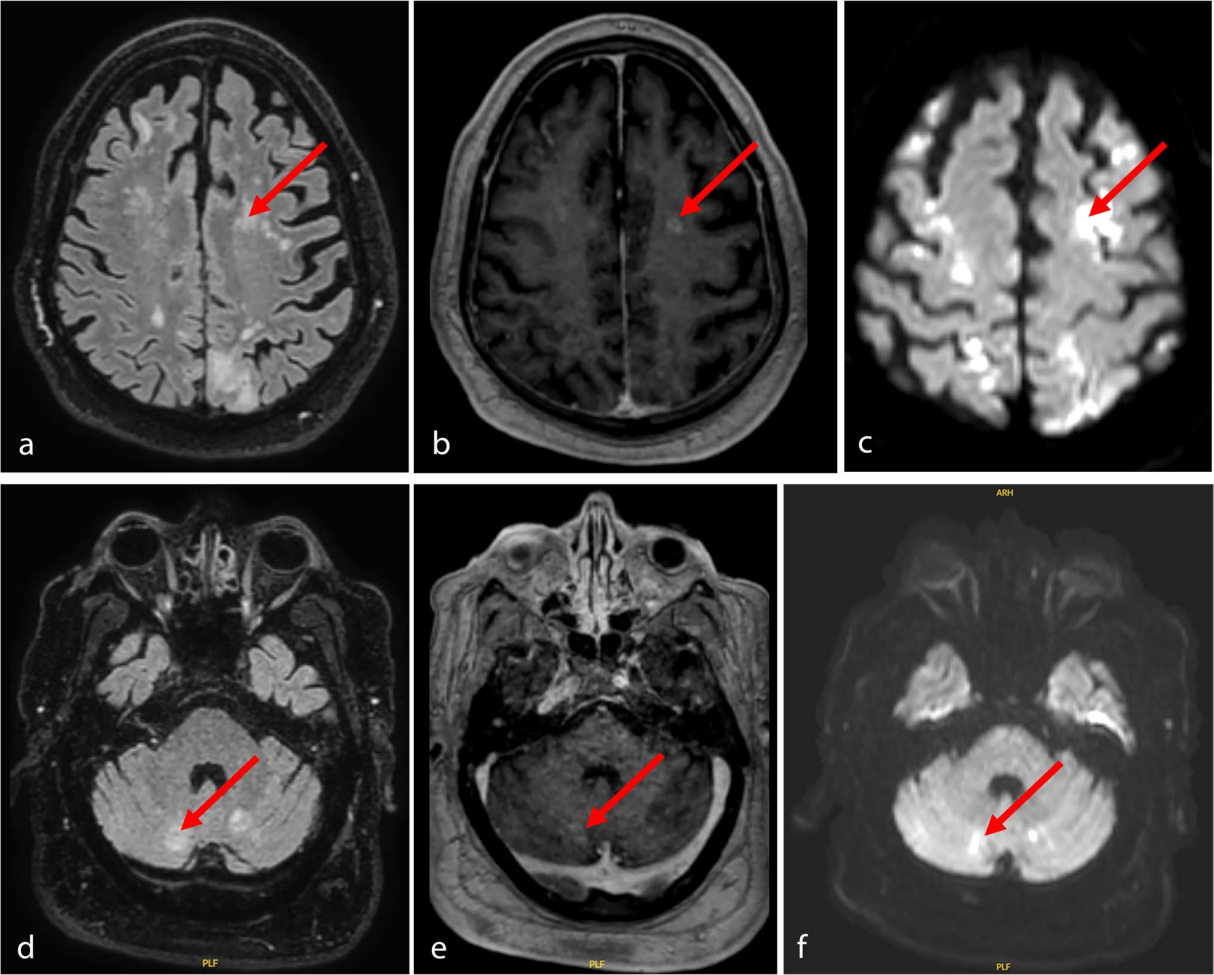
Axial cranial MRI one day after petroleum exposition. Imaging revealed both supratentorial (upper row) and cerebellar (lower row) FLAIR hyperintense (**a** and **d**), T1-Gadolineum-enhancing (**b** and **e**) diffusion-restricted (**c** and **f**) lesions, suspicious for cytotoxic edema (red arrows, each). In contrast, contrast-enhancing lacunar infarctions were considered unlikely due to the short duration between exposition/onset of the symptoms and imaging

A 75-year-old male was transferred to our hospital after accidental lumbosacral infiltration of 4 ml petroleum, mistaken for cortisone and intended to be used for disinfection for an orthopedic procedure due to sciatica. The patient presented with localized lumbar pain at the site of infiltration, new onset of paresthesia of the lower right extremity as well as with arterial hypertension, tachypnea, and impaired oxygenation. The infiltration site on the skin could not be located. A computed tomography scan revealed lumbosacral intramuscular and intraspinal gas, likely due to evaporation and bubble formation, with ventral and cranial distribution along the lumbar nerve roots (Fig. 1), pulmonal ground glass opacity and small bilateral pleura effusions. According to the gas distribution, petroleum diffusion into the thecal sac was assumed, while primary intrathecal injection appeared unlikely according to its intended use and the obese constitution of the patient. Following consultation of the regional toxicology center, emergency local irrigation was indicated, and the patient was immediately transferred to the operating room (OR) 3 h following exposition. Intraoperatively, lumbosacral soft and muscle tissue appeared physiological, and extensive irrigation was performed. The dural sac was exposed through an interlaminar approach and appeared intact. A lumbosacral soft tissue drain was placed, and the patient was transferred to the intensive care unit (ICU) for surveillance. Sixteen hours after drug exposition, the patient developed tetraparesis and sopor. Subsequent spinal magnetic resonance imaging (MRI) revealed no relevant spinal pathology, while cranial imaging showed several cerebellar and supratentorial FLAIR (fluid-attenuated inversion recovery) -hyperintense, diffusion-restricted lesions displaying weak contrast-enhancement (Fig. 2), possibly indicating intrathecal petroleum diffusion and tissue reactions. The patient was transferred to the OR, where both a right frontal external ventricular as well as a lumbar drain were inserted. Postoperatively, the patient developed both hemodynamic and respiratory deterioration, and progressive bilateral pleura effusions were treated by additional drains. Subsequently, 100 ml/h lactate-free ringer solution was administered through the ventricular drain using a perfusor system under constant monitoring of the intracranial pressure, and equivalent amounts of fluid were drained lumbar. This constant irrigation was maintained under strict observation of fluid in- and output for 16 h, and the patient additionally received 1 g methylprednisolone. Subsequent repeated analyses of the cerebrospinal fluid (CSF) revealed hyperglycemia (137 mg/dl) but no other relevant findings. Due to persistent coma, electroencephalography (EEG) was performed revealing severe deceleration with no signs of seizures. Serum neuron-specific enolase (NSE) 3 days after exposition was increased (33.2 ng/ml (0–16.3) with a similar intrathecal concentration (33.3 ng/ml), and serum creatine kinase (CK) remained moderately elevated (1600U/ml). After the exclusion of hydrocephalus, the ventricular drain was removed. Follow-up EEG displayed persisting, but improved decelerations and the patient’s general condition improved slowly. Further MRIs confirmed the previously described cranial lesions, suspicions for either cytotoxic edema. Fifteen days after the exposition, the patient was discharged to rehabilitation being awake, able to speak and moving both upper and lower limbs.

## Discussion

Parenteral injection of petroleum, e.g., intrapleural [2] or into soft tissue [5, 7, 10], has been reported in a few cases and is usually performed in the context of attempted suicide. Similar agents, such as petroleum ether (Naphta), are commonly used for the perioperative cleaning of surgical sites. Due to its distinct lipophilic character, petroleum easily penetrates (soft) tissue, leading to an extensive distribution and severe local necrosis or even fasciitis. In our case, petroleum was accidentally used for intended *local* lumbosacral infiltration. Hence, due to the obesity of the patient, the lack of a dura tear intraoperatively, and the distribution into the pelvis, secondary diffusion might have led to intrathecal distribution, while primary intradural injection appeared unlikely. However, the distinct dispensation following 2 h after injection is noteworthy. Similarly, the discrete local tissue reaction on initial imaging is remarkable, but might be explained by the limited capability of CT to display soft tissue. According to previous reports, local irrigation was performed and prevented further soft tissue necrosis as demonstrated by subsequent MRI and a moderately elevated serum CK level. Of note, the patient nevertheless developed delayed neurological deterioration following 16 h after the exposition, indicating further central nervous system distribution of the petroleum. Imaging findings, elevated serum and CSF NSE levels, severeness of the symptoms as well as EEG findings were strongly suggestive for ascending, petroleum-induced myeloencephalopathy. In fact, cranial imaging characteristics were suggestive of local inflammation and disruption of the blood–brain barrier but not typical for ischemic infarction. In contrast to lumbar intrathecal irrigation occasionally reported after unintended intrathecal administration of local anesthesia [6, 11], systemic irrigation after establishing an external ventricular drain is rarely described and only after accidental administration of vincristine [1, 8, 9]. Here, the beginning of lavage therapy ranged from immediately to 24 h after drug administration and was performed with 0.9% saline or ringers lactate [9], mostly with similar rates (50–100 ml/h) as used in our case. In contrast, most cases following intrathecal vincristine administration not treated with CSF lavage displayed fatal outcome [9]. Among survivors, the outcome after CSF lavage varied, presumably also depending on the administered drug and dosage. However, independent of the applied drug, intrathecal lavage itself appears a safe and feasible option for the treatment in these critical patients but requires strict observation of intracranial pressure and fluid in- and output. As petroleum likely causes severe, ascending myeloencephalopathy even after secondary intrathecal diffusion, combined local and CSF irrigation appears a safe and reasonable therapy approach in these otherwise complicated, critically ill patients. Of note, the natural course of the disease is obscure, so the impact of the applied therapy on symptom improvement finally remains unclear.

Numerous causes for pulmonal ground glass opacity have been described, including infections, neoplasms and exposures [4]. As already present in CT scans only few hours after lumbar petroleum injection, pre-existence in our case has been discussed. However, distinct progression of both ground glass opacity and pleura effusions on follow-up scans 28 h later (images not shown) and the lack of history of pulmonal disease suggest an association with petroleum injection, e.g., in terms of a pneumonitis.

Due to its lipophilic character, petroleum might penetrate the dura secondary to lumbar infiltration and leads to myeloencephalopathy and severe delayed neurological symptoms. Aside from local surgical revision, the establishment of cranial and lumbosacral drains appears a feasible option to allow continuous CSF irrigation and might help to reduce subsequent neurological symptoms.

## Data Availability

Data can be provided by the corresponding authors upon request.
